# Leveraging Guideline-Based Clinical Decision Support Systems with Large Language Models: A Case Study with Breast Cancer

**DOI:** 10.1055/a-2528-4299

**Published:** 2025-04-16

**Authors:** Solène Delourme, Akram Redjdal, Jacques Bouaud, Brigitte Seroussi

**Affiliations:** 1 Sorbonne Université, Université Sorbonne Paris Nord, INSERM, LIMICS, Paris, France; 2 EPITA, Paris, France; 3 Univ Gustave Eiffel, Aix-Marseille Univ, LBA, Marseille, France; 4 Sorbonne Université, AP-HP, Tenon Hospital, Public Health Department, INSERM, Université Sorbonne Paris Nord, Limics, Paris, France; 5 APREC, Paris, France

**Keywords:** clinical decision support systems, OncoDoc2, breast cancer, large language models, question-answering systems

## Abstract

**Background** Multidisciplinary tumor boards (MTBs) have been established in most countries to allow experts collaboratively determine the best treatment decisions for cancer patients. However, MTBs often face challenges such as case overload, which can compromise MTB decision quality. Clinical decision support systems (CDSSs) have been introduced to assist clinicians in this process. Despite their potential, CDSSs are still underutilized in routine practice. The emergence of large language models (LLMs), such as ChatGPT, offers new opportunities to improve the efficiency and usability of traditional CDSSs.

**Objectives** OncoDoc2 is a guideline-based CDSS developed using a documentary approach and applied to breast cancer management. This study aims to evaluate the potential of LLMs, used as question-answering (QA) systems, to improve the usability of OncoDoc2 across different prompt engineering techniques (PETs).

**Methods** Data extracted from breast cancer patient summaries (BCPSs), together with questions formulated by OncoDoc2, were used to create prompts for various LLMs, and several PETs were designed and tested. Using a sample of 200 randomized BCPSs, LLMs and PETs were initially compared with regard to their responses to OncoDoc2 questions using classic metrics (accuracy, precision, recall, and F1 score). Best performing LLMs and PETs were further assessed by comparing the therapeutic recommendations generated by OncoDoc2, based on LLM inputs, to those provided by MTB clinicians using OncoDoc2. Finally, the best performing method was validated using a new sample of 30 randomized BCPSs.

**Results** The combination of Mistral and OpenChat models under the enhanced Zero-Shot PET showed the best performance as a question-answering system. This approach gets a precision of 60.16%, a recall of 54.18%, an F1 score of 56.59%, and an accuracy of 75.57% on the validation set of 30 BCPSs. However, this approach yielded poor results as a CDSS, with only 16.67% of the recommendations generated by OncoDoc2 based on LLM inputs matching the gold standard.

**Conclusion** All the criteria in the OncoDoc2 decision tree are crucial for capturing the uniqueness of each patient. Any deviation from a criterion alters the recommendations generated. Despite achieving a good accuracy rate of 75.57%, LLMs still face challenges in reliably understanding complex medical contexts and be effective as CDSSs.

## Introduction

The International Agency for Research on Cancer (IARC) predicts a 77% increase in cancer cases between 2022 and 2050. 1 As the incidence rises, the management of breast cancer is becoming increasingly complex. Multidisciplinary tumor boards (MTBs) have been established to support therapeutic decision-making for cancer patients. However, they are often confronted with clinical case overloads and limited discussion time, which affect the quality of care. Clinical decision support systems (CDSSs) have proven to be effective tools to assist clinicians in their decision-making process. 2 When based on clinical practice guidelines (CPGs), CDSSs provide patient-centered therapeutic recommendations aligned with guidelines. However, despite their potential to improve the compliance of MTB decisions with CPGs, many guideline-based CDSSs remain underutilized, and only few have successfully been integrated into routine clinical practice due to technical or usability barriers. 3

OncoDoc 4 and OncoDoc2 5 are decision support systems for the management of breast cancer, based on a documentary approach. Clinicians navigate through a knowledge base structured as a decision tree, answering questions to describe a patient's personal and familial antecedents, general state, and tumor characteristics, to finally obtain patient-specific recommendations based on Cancer Est guidelines. 5 OncoDoc2 has been routinely used at the Tenon Hospital (Assistance Publique – Hôpitaux de Paris, Paris, France) between February 2007 and October 2009 showing a 91.7% compliance of Tenon MTB decisions with OncoDoc2 across approximately 2,000 decisions. 5 However, usability issues with OncoDoc2 have finally hindered its routine use as a CDSS.

The emergence of large language models (LLMs), such as OpenAI's ChatGPT in 2022, has opened new possibilities for enhancing CDSSs. LLMs are artificial intelligence (AI) algorithms based on deep neural network architectures like Transformers. 6 Trained on vast amounts of textual data, these models are able to generate text with varying degrees of coherence and contextual relevance. Despite their promise, LLMs currently face limitations in understanding highly specialized medical contexts, often generating outputs that look syntactically coherent but may lack of medical semantic accuracy. This reliability gap is a critical barrier to their application in clinical settings. In addition to accuracy, sustainability plays a key role in the selection and evaluation of LLMs, particularly in healthcare settings as computational efficiency can preserve the planet's natural resources and reduce operational costs.

Nevertheless, the capabilities of LLMs for analyzing and synthesizing large volumes of textual data can be particularly relevant in oncology, where the volume of information is substantial, and the personalization of treatments is crucial. 7 Thus, LLMs could enhance CDSSs, if their outputs are carefully evaluated to ensure they provide medically sound recommendations. In the case of OncoDoc2, LLMs may offer the opportunity to automatically answer the CDSS questions based on patient records and summaries, thus automating the navigation through the decision tree, potentially increasing the system's usability. Yet, the extent to which LLMs can accurately interpret the specific medical context of each patient and provide reliable, patient-centered recommendations remains an open question.

To test this hypothesis, we developed and evaluated a question-answering (QA) system based on open-access LLMs to automate OncoDoc2 decision support process for breast cancer treatment. The aim was to generate best patient-specific therapeutic recommendations by integrating OncoDoc2 decision tree within the LLM reasoning process, based on breast cancer patient summaries (BCPSs), streamlining the decision-making process for MTB clinicians. Through this work, the aim was to use LLMs to improve an existing CDSS while addressing the following research questions:

1. Can LLMs accurately navigate OncoDoc2 decision tree based on patient data extracted from BCPSs?
2. How effective are different prompting techniques in enhancing LLM performance for clinical decision-making?
3. What are the limitations and future possibilities for integrating LLMs into guideline-based CDSSs to enhance the quality of care for breast cancer patients?

## Materials

### OncoDoc2

**Fig. 1:**
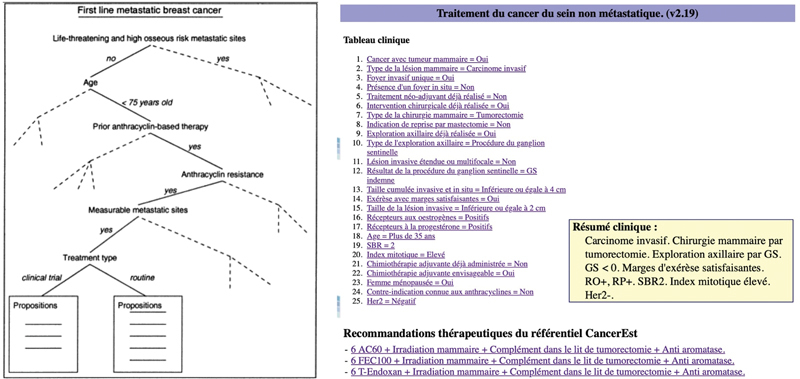
OncoDoc2 decision tree and navigation via the OncoDoc2 user interface.

OncoDoc is a guideline-based CDSS designed for the management of breast cancer patients. 4 Developed in a documentary paradigm, it allows users to interactively navigate its knowledge base structured as a decision tree (see Fig. 1 ). By answering questions that characterize a patient's specific condition, users can obtain tailored therapeutic recommendations. The decision tree includes 69 clinical parameters (nodes) corresponding to decision variables. Arcs of the decision tree correspond to the values of the clinical parameters. The leaves of the tree give the treatment proposals recommended for the patient profiles represented by the paths through the decision tree, leading to the leaves. The later version of OncoDoc, OncoDoc2, has been extended to provide an enriched decision tree made of 2,305 possible paths, covering therapeutic decisions according to clinical practice guidelines (CPGs) for non-metastatic breast cancer. We used the set of 1,886 decisions made between February 2007 and October 2009, when OncoDoc2 was routinely used at the Tenon hospital (Assistance Publique – Hôpitaux de Paris, Paris, France). Decisions were summarized in an Excel spreadsheet of 1,886 rows. Each row represented the decision made for patient cases discussed during MTB meetings (see an example in Appendix 1 , available in the online version) and described in 69 attributes. The XML representation of OncoDoc2 decision tree, consisting of the 69 clinical attributes and 2,305 paths, was used to make the LLM automatically navigate the decision tree until getting recommendations, which we compared with the recommendations made by MTB clinician navigating for the same patient cases, considered as the gold standard.

The study where OncoDoc2 has been used in the MTB of Tenon hospital was declared to the Comité Consultatif de Protection des Personnes en matière de Recherche Biomédicale (Institutional Review Board) of Saint-Antoine Hospital in Paris, as well as to the Commission Nationale de l'Informatique et des Libertés (French Data Protection Authority), ensuring compliance with all applicable legal and ethical standards. In line with institutional policies of Greater Paris University Hospitals (AP-HP), patients were informed that their health data could be reused for research purposes and were made aware of their right to object to such reuse.

### Breast Cancer Patient Summaries

**Fig. 2:**
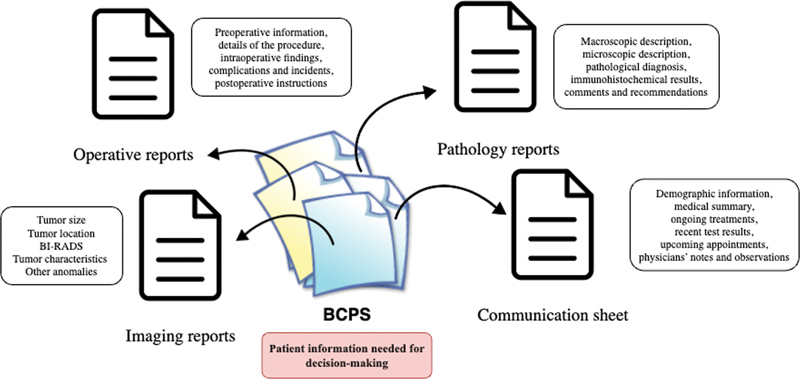
Building breast cancer patient summary (BCPS) content from different reports of a patient's electronic health record (EHR).

BCPSs are narrative natural language documents that summarize the clinical situation of a breast cancer patient, describe the reasoning process to establish the cancer diagnosis, and provide the collective decision made by MTB clinicians. BCPSs contain information about the patient, examination results, tumor characteristics, and the treatments already received. An example of a BCPS is shown in Fig. 2 . The information necessary to accurately describe a clinical case should exist in BCPSs. However, it happens that BCPSs are incomplete or inconsistent, making the task of extracting relevant information from BCPSs even more challenging. Besides, each BCPS is unique, making the overall information extraction process complex.

For this study, we used a set of 230 BCPSs randomly selected from among the set of BCPSs for which the MTB clinician navigated through OncoDoc2 available in the provided Excel file (see section 3.1, Selection of LLMs for Question Answering). BCPSs were retrieved from the Tenon Hospital data warehouse (Assistance Publique – Hôpitaux de Paris, Paris, France). The dataset included cases treated between February 2007 and October 2009. Among the 230 BCPSs, 200 were randomly selected for the evaluation (S ^Evaluation^ ) and the 30 remaining BCPSs were used for the system validation (S ^Validation^ ). Among S ^Evaluation^ , a sample of 20 randomly selected BCPSs were used for the model selection (S ^Selection^ ).

### Large Language Models (LLMs)

Since BCPSs contain personal health data, we chose to use open-source LLMs that may be processed locally to ensure that personal health data are not sent to external servers (as it is the case with ChatGPT), thereby guaranteeing the confidentiality and security of this sensitive data. As (i) Orca and Gemma have been trained on medical data, 8 9 10 (ii) OpenChat has been trained on raw unstructured data, 11 (iii) Llama has proven its effectiveness as a question-answer system, 9 and (iv) Mistral has shown good performance in real-world applications, 12 we chose to work with Orca 7B 8 (Microsoft), Llama 7B 9 (Meta), Gemma 7B 10 (independent researchers), Mistral 7B, 12 and OpenChat 7B 11 (independent researchers). Additionally, we tested a combination of models to assess potential performance gains. We also tested Mixtral 8 × 7B 13 on the best prompt engineering technique (PET) studied to test the performance of a larger model.

## Methods

**Fig. 3:**
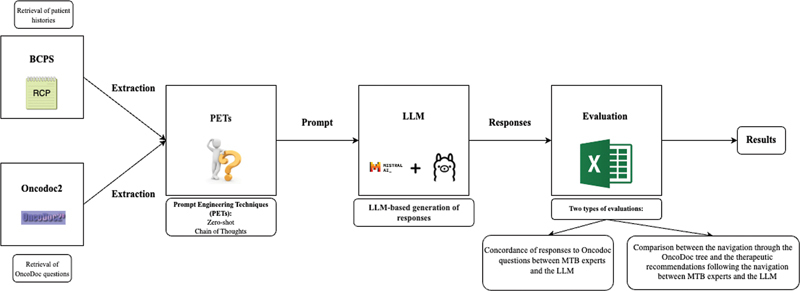
Workflow illustrating the method implemented.

Fig. 3 illustrates the methodology we implemented to develop the Q/A system, working with BCPSs and OncoDoc2. The process involves retrieving data from BCPSs, creating prompts for LLMs, and generating responses with OncoDoc2. We conducted a two-step evaluation by (i) comparing LLM and MTB clinician answers to characterize a clinical case, and (ii) comparing the treatment recommendations generated for the clinical case by LLMs and MTB clinicians using OncoDoc2.

**Fig. 4:**
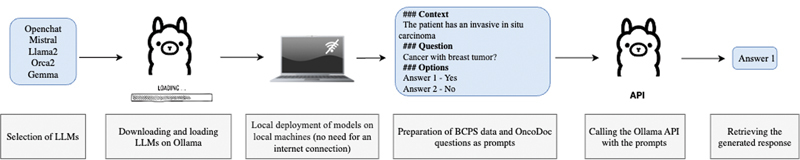
Ollama operational flowchart.

1. **Data retrieval** : The first step was to retrieve the navigations performed by clinicians during MTBs and to gather all the questions asked by OncoDoc2 to compare MTB clinician navigations with the navigations generated by LLMs. We also collected data from BCPSs (patient history, pathology data, operative information, etc.) to provide the necessary context for LLMs to navigate.
2. **Creation of instructions for LLMs** : With the retrieved information, we created the instructions for LLMs. Various PETs have been used to guide the model toward the expected answers. The instructions, in French language, include the patient's history, the question asked, and possible answer options.
3. **Generation of LLM responses** : Instructions were provided to the various LLMs, and the generated texts were processed to get LLM responses to be used to evaluate the method (see Fig. 4 ).
4. **Two-step evaluation of LLM performance:**
  - First, for each Oncodoc2 question, we assessed the concordance of LLM responses with the responses given by MTB clinicians for the same questions (details in Section 3.3, Evaluation of LLMs Used as Question-Answering Systems) considered as the gold standard (GS). We used accuracy, precision, recall, and F1 score to evaluate LLM's performance as question/answer systems.
  - Then we evaluated LLM's ability to provide correct therapeutic recommendations to a given patient clinical case described by her BCPS by comparing the recommendations proposed by OncoDoc2 following the MTB clinician navigation to those proposed by OncoDoc2 following the LLM navigation for the same patient.

Each LLM was first downloaded and loaded onto the Ollama platform 14 to allow the model to be deployed locally without additional configuration. In this way, the model's execution did not require an internet connection and security of health data was guaranteed. We then used the Ollama API to retrieve the responses generated by the different LLMs. Fig. 4 shows the pipeline developed.

### Selection of LLMs for Question Answering

To select the LLMs to work with, we tested five different LLMs, namely, Mistral, OpenChat, Orca2, Gemma, and Llama (cf. Section 2.3, Large Language Models (LLMs)), on their ability to answer the OncoDoc2 questions and on their ecological impact. We used the sample of 20 BCPSs randomly selected (S ^Selection^ ) from the 200 used for model evaluation (S ^Evaluation^ ). For each BCPS, we used a set of questions from OncoDoc2 decision tree. The prompts were executed twice to verify the model's reproducibility and the results were compared with the answers provided by MTB clinicians for each question.

We also considered the size of the five models to assess their overall ecological impact, and measured the carbon footprint of LLM computations on the 20 BCPSs of S ^Selection^ , particularly when using LLMs to generate responses to OncoDoc2 questions (we used a Python package called CarbonTracker 15 ).

We finally selected the two models that demonstrated the highest performance while maintaining the lowest environmental impact, denoted BPM1 and BPM2, for the training and evaluation phases of the analysis.

### Prompt Engineering

The following prompting engineering techniques (PETs) were applied to evaluate LLM's performance:

- **Zero-Shot technique**^16^ (see Appendix 2 , available in the online version) involves direct instruction without providing any example to the model (e.g., “Cancer with breast tumor? Yes/No”).
- **Enhanced Zero-Shot technique** (see Appendix 3 , available in the online version) refines the formulation of questions and answer options from OncoDoc2 decision tree without providing examples. For instance, the question: “Cancer with breast tumor? with option 1: Yes, and option 2: No” becomes: “Does the patient have a cancer with a breast tumor? with option 1: Yes, the patient has a cancer with a breast tumor and option 2: No, the patient does not have a cancer with a breast tumor.”
- **Zero-Shot Chain-of-Thought technique (Zero-Shot CoT)**^17^ (see Appendix 4 , available in the online version) encourages the model to follow a series of reasoning steps before providing an answer. This allows the model to structure its reasoning process more logically and coherently (e.g., “Based on pathology data, determine if cancer is present, then decide if the tumor is a breast tumor, think step by step”).

### Evaluation of LLMs Used as Question-Answering Systems

**Fig. 5:**
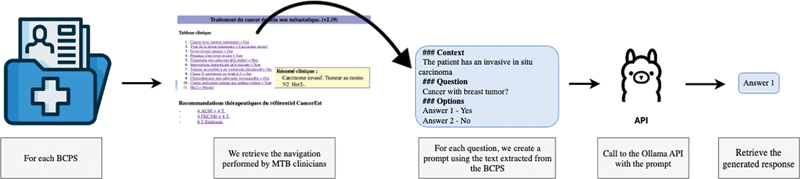
Large language model (LLM) used as a Q/A system based on OncoDoc2 decision tree.

Using the two best-performing models (BPM1 and BPM2), we automated the method presented in section 3.1 (Selection of LLMs for Question Answering) (see Fig. 5 ) for the 200 BCPSs of S ^Evaluation^ . For each question, the answer of LLMs was compared with the answer given by MTB clinicians for the same question, and the same clinical patient case, at the moment the case was discussed. We evaluated the accuracy for all questions for all BCPSs. The results were categorized into three groups: accuracy above 80% ( **high** ), between 60 and 80% ( **average** ), and below 60% ( **low** ). Based on this evaluation, we kept the best-performing prompt technique for each question. The enhanced Zero-Shot technique was only used when Zero-Shot accuracy was below 100%. For questions where the accuracy was low for both BPM1 and BPM2, we used the Zero-Shot CoT technique to attempt improvement. We also evaluated the Zero-Shot CoT technique across all BCPSs to assess its overall performance.

### Evaluation of LLMs Used as Decision Support Systems

**Fig. 6:**
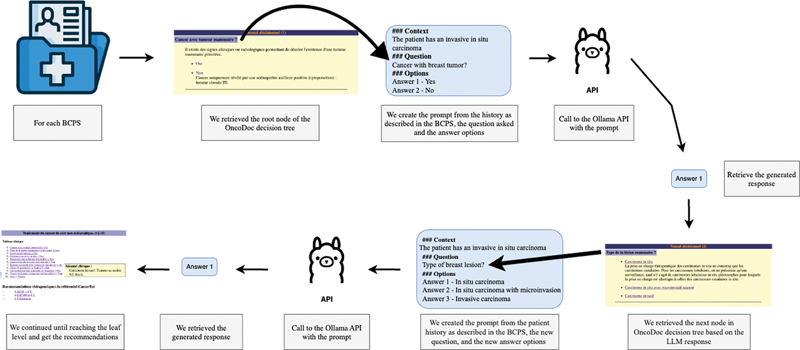
Large language model (LLM) used as a clinical decision support system (CDSS) based on OncoDoc2 decision tree.

Building on the previous evaluation, the top-performing LLMs and PETs were applied to the 200 BCPSs from S ^Evaluation^ to assess how accurately the LLMs could generate appropriate therapeutic recommendations (as illustrated in Fig. 6 ). We supplied the LLMs with BCPSs and the root question of OncoDoc2 decision tree. Based on the responses generated at each level from LLM inputs, the system navigated through OncoDoc2, progressing to the leaf level to obtain therapeutic recommendations. The evaluation focused on comparing the recommendations issued by the navigation performed by the LLM (denoted Recos ^LLM^ ) to the recommendations issued from the navigation performed by MTB clinicians considered as the gold standard (denoted Recos ^GS^ ). We distinguished three main situations:

- When Recos ^LLM^ = Recos ^GS^ , then **Conf (Recos ^LLM^ , Recos ^GS^ ) = identical** .
- When Recos ^LLM^ ≠ Recos ^GS^ but the care plans of both Recos ^LLM^ and Recos ^GS^ were made of the same treatment modalities (surgery, radiotherapy, hormone therapy, chemotherapy, or targeted therapies), AND were organized in the same order, we explored each proposed treatment modalities.
○ For surgery, we proposed a classification of surgery modalities (see Appendix 5 , available in the online version) and an expert oncologist specified which surgery modalities could be considered as comparable.
○ For other modalities (radiotherapy, hormone therapy, chemotherapy, and targeted therapies), we considered that they were comparable as long as the modality in Recos ^LLM^ was subsumed by the modality in Recos ^GS^ .
When all treatment modalities in the LLM's proposal were *comparable* to those of the gold standard and proposed in the same order, then we considered the recommendations were comparable, i.e., **Conf (Recos ^LLM^ , Recos ^GS^ ) = comparable.
**
- When recommendations were neither identical nor comparable, then **Conf (Recos ^LLM^ , Recos ^GS^ ) = different.
**

### Validation

**Fig. 7:**
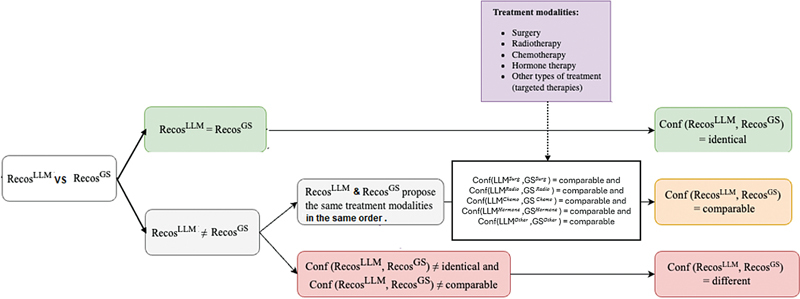
Comparison of the leaves of large language model (LLM) navigations with multidisciplinary tumor board (MTB) clinician navigations, and evaluation of recommendation conformity.

Validation was performed using the reserved 30 BCPSs of S ^Validation^ . For each case, LLM inputs were compared with MTB-derived navigations. We conducted the validation of LLMs as question-answering systems and as decision support systems using a combination of BPM1 and BPM2 in an enhanced Zero-Shot framework, where the models were used together while selecting the best-performing model for each question asked during the navigation throughout OncoDoc2 decision tree, based on their results on the evaluation dataset ( Fig. 7 ).

## Results

### Selection of LLMs

**Fig. 8:**
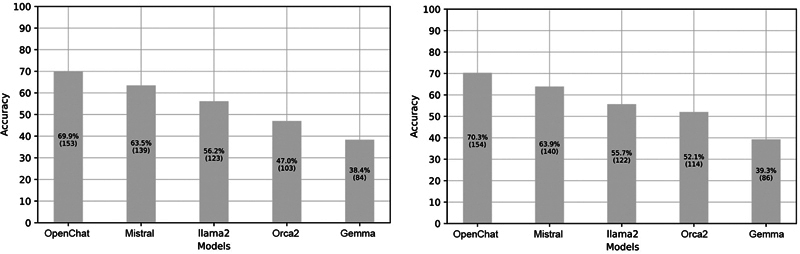
Accuracy of the five models in Zero-Shot prompt engineering technique (PET) on S ^Selection^ , first trial on the left and second trial on the right.

**Fig. 9:**
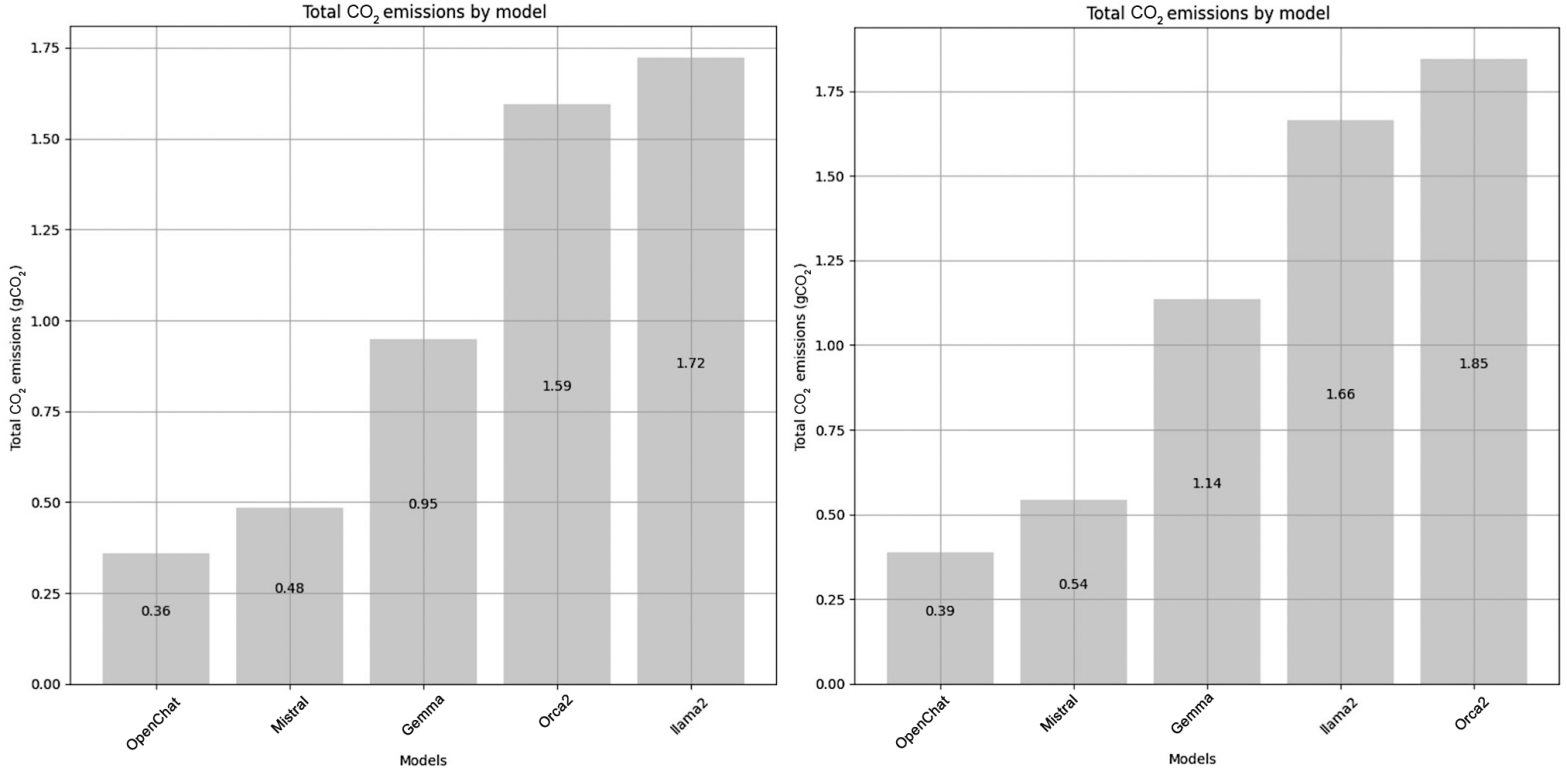
Total CO _2_ equivalent in grams for the five models on S ^Selection^ , first trial on the left and second trial on the right.

Fig. 8 presents the performance of each of the five models with Zero-Shot PET to evaluate the best LLM on the sample of 20 BCPSs of S ^Selection^ . Models are displayed by accuracy computed on a total of 220 questions. The number of correct answers is indicated on each bar. Results show that Mistral and OpenChat are the best-performing models with an accuracy of 63.5 to 63.9% and 69.9 to 70.3% respectively. Fig. 9 illustrates the ecological impact of the five models on the same BCPSs. Again, Mistral and OpenChat demonstrated the lowest CO _2_ emissions, with Mistral's impact ranging from 0.48 gCO2eq to 0.54 gCO2eq and OpenChat ranging from 0.36 gCO2eq to 0.39 gCO2eq. Based on these results, Mistral (BPM1) and OpenChat (BPM2) were selected for the following evaluations in this study.

### Comparison of Prompt Engineering Techniques

We worked on the 200 randomly selected BCPSs of the evaluation sample S ^Evaluation^ , corresponding to a total of 3,142 prompts concerning the 69 questions of OncoDoc2 decision tree. We analyzed various PETs on Mistral and OpenChat, the Zero-Shot, the Zero-Shot Chain-of-Thought, and the enhanced Zero-Shot for Mistral 7B and OpenChat models. For the enhanced Zero-Shot PET, which yielded the best results, we also used Mixtral 8 × 7B (a larger model with more parameters). Table 1 presents the results of the different PETs used on the three models, based on precision, recall, F1 score, and accuracy metrics.

**Table 1:** Results of the different PETs with Mistral, Mixtral 8 × 7B, and OpenChat models on 3,142 prompts

PET	Model	Precision (%)	Recall (%)	F1 score (%)	Accuracy (%)
**Zero-Shot**	*Mistral*	54.27	49.34	47.44	61.78
	*OpenChat*	**72.22**	**70.73**	**71.30**	**69.95**
**Enhanced Zero-Shot**	*Mistral*	57.26	47.67	49.62	66.10
	*Mixtral8 × 7B*	61.69	58.06	59.45	**77.08**
	*OpenChat*	**73.54**	**71.31**	**72.11**	72.47
**Zero-Shot CoT**	*Mistral*	54.80	44.27	45.70	59.83
	*OpenChat*	55.74	49.13	51.60	**69.96**

Abbreviation: PET, prompt engineering technique.

The simple Zero-Shot PET showed variable results, with OpenChat achieving a precision of 72.22% and an accuracy of 69.95%, significantly outperforming Mistral (with 54.27% and 61.78%, respectively). The enhanced Zero-Shot PET enhanced models' performance: Mixtral 8 × 7B stands out with an accuracy of 77.08% (which may be explained by the fact it is the largest model), and OpenChat achieved the best overall performance with a precision of 73.54% and an accuracy of 72.47%. Among the evaluated techniques, enhanced Zero-Shot consistently outperformed Zero-Shot and Zero-Shot CoT in precision, recall, and F1 score, indicating its superiority to disambiguate questions and guide models. Zero-Shot CoT, while intended to encourage logical reasoning, underperformed in simpler questions due to hallucination effects, reducing accuracy.

### Assessment of LLMs Used as Question-Answering Systems

We worked on the same sample of 200 randomly selected BCPSs of S ^Evaluation^ . We analyzed the distribution of all 69 questions used to navigate the OncoDoc2 decision tree with the enhanced Zero-Shot prompt engineering (PE), previously identified as the most effective technique based on the accuracy achieved during the training phase step (see Table 2 ).

**Table 2:** Accuracy percentages and number of questions for each accuracy category for OpenChat and Mistral models

Model	<60%	Number	60–80%	Number	>80%	Number	Total
**Mistral**	36.20	**25**	24.60	17	39.10	**27**	69
**OpenChat**	33.33	23	36.23	**25**	30.43	21	69

#### LLM Accuracy on OncoDoc2 Questions

**Fig. 10:**
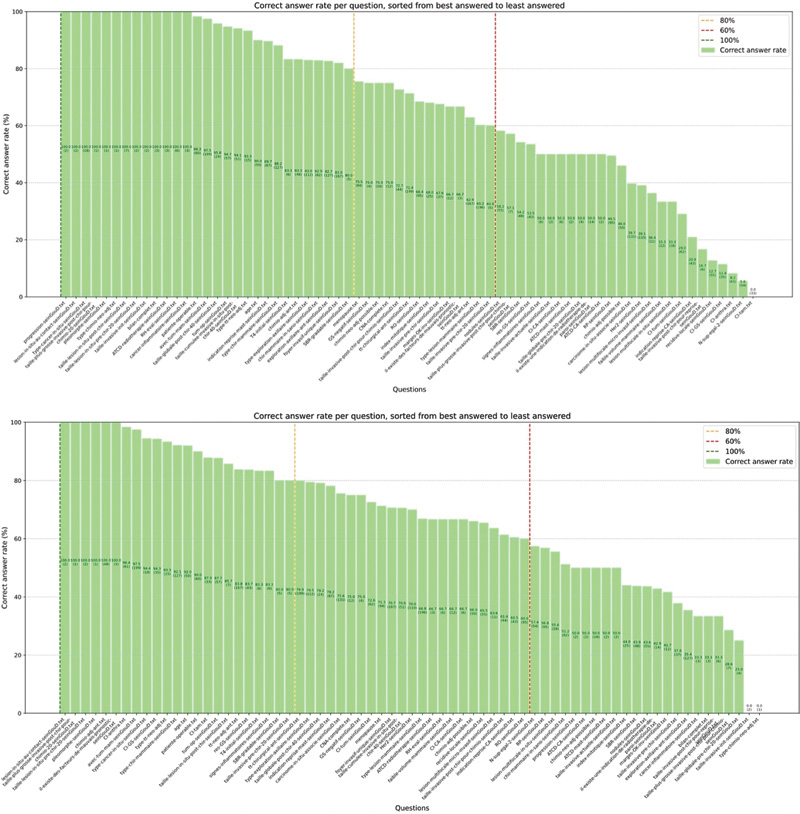
Distributions of Mistral (top) and OpenChat (bottom) models' accuracy to answer OncoDoc2 questions with the enhanced Zero-Shot prompt engineering technique (PET).

Fig. 10 presents models' accuracy in answering OncoDoc2 questions with the percentages of correct answers, categorized into three groups, high, average, and low (ranges 60, 80, and 100%). This distribution illustrates the performance of Mistral and OpenChat models, with the enhanced Zero-Shot PET (larger versions of figures are presented in Appendix 6 and Appendix 7 (available in the online version), and Zero-Shot CoT distributions for both models are presented in Appendixes 8 and 9 , available in the online version).

The enhanced Zero-Shot PET results with Mistral are quite variable. For example, for the question about the presence of a breast tumor, the model achieves 97.49% of correct answers (194/200 BCPSs) but drops to 4.9% for the question about the existence of anthracyclin contraindications (3/61 BCPSs). OpenChat demonstrated higher overall accuracy compared with Mistral, e.g., achieving 98.40% accuracy for anthracyclin contraindications (60/61 BCPSs). However, Mistral excelled in specific questions, with 27 questions (nodes of OncoDoc2 decision tree) achieving >80% accuracy, highlighting its capacity to better handle nuanced scenarios than OpenChat, even though more than half of these 27 questions were actually rarely asked (14/27 questions were asked on less than 10 BCPSs with a performance above 80%).

#### Choosing the Best Model for Each OncoDoc2 Question

Table 3 presents the results of the enhanced Zero-Shot and Zero-Shot CoT PETs, combining both Mistral and OpenChat models, based on their performance on OncoDoc2 questions as previously obtained (see Fig. 10 ). Questions were assigned to each model based on their respective performance to maximize the overall efficiency. Table 3 shows that both enhanced Zero-Shot and Zero-Shot CoT PETs achieved quite similar scores in terms of precision, recall, F1 score, and accuracy.

**Table 3:** Results of the different PETs combining Mistral and OpenChat models based on their performance on OncoDoc2 questions

PETs	Precision (%)	Recall (%)	F1 score (%)	Accuracy (%)
**Enhanced Zero-Shot**	59.05	54.40	56.35	77.05
**Zero-Shot CoT**	61.06	53.48	56.30	75.87

Abbreviation: PETs, prompt engineering techniques.

### Assessment of LLMs Used as Decision Support Systems

We worked on S ^Evaluation^ to compare the recommendations generated by Mistral and OpenChat (Recos ^LLM^ ) to the recommendations obtained by MTB clinicians (Recos ^GS^ ) (see Section 3.4, Evaluation of LLMs Used as Decision Support Systems) and assess whether, based on LLM inputs, the provided recommendations were identical, comparable, or different from the GS. The results of this comparison are presented in Table 4 . We compared two PETs:

- The enhanced Zero-Shot PET using Mistral alone, OpenChat alone, and the combination of both models.
- The Zero-Shot CoT with the combination of Mistral and OpenChat.

**Table 4:** Percentage of compliant recommendations produced by LLM navigation (identical, comparable, different) as compared with those produced by MTB clinician navigations

PET	Model	Identical (%)	Comparable (%)	Different (%)
**Enhanced Zero-Shot**	*Mistral*	11.44	14.42	74.12
	*OpenChat*	9.00	17.91	73.13
	*Mistal & OpenChat*	**17.91**	**19.40**	**62.68**
**Zero-Shot CoT**	*Mistal & OpenChat*	7.46	14.92	79.10

Abbreviations: LLM, large language model; MTB, multidisciplinary tumor board.

For the enhanced Zero-Shot PET, the combination of Mistral and OpenChat achieved better results compared with using the models separately, with 17.91% identical recommendations, 19.40% comparable recommendations, and 62.68% different recommendations. In contrast, the Zero-Shot CoT PET with the combined models produced 7.46% identical recommendations, 14.92% comparable recommendations, and 79.10% of recommendations categorized as different.

### Validation

The validation phase was conducted on the sample of 30 BCPSs, randomly selected for validation (S ^Validation^ ). We first evaluated the performance of the combination of Mistral and OpenChat as a Q/A system using the enhanced Zero-Shot PET on OncoDoc2 questions (as represented in Table 5 ). Then we assessed LLM's ability to navigate OncoDoc2 and generate recommendations (as represented in Table 6 ). The similarity in results of Table 5 and Table 6 confirms that LLMs performed reasonably well as question-answering systems, with a precision of 60.16%, recall of 54.18%, F1 score of 56.59%, and accuracy of 75.57%. However, used as decision support systems, they provided poor results, with only 3.34% identical recommendations, 13.33% comparable, and 83.33% different recommendations. These results underscore the challenges of aligning LLM-driven navigations with MTB clinician practices, particularly in cases requiring nuanced contextual understanding.

**Table 5:** Results of enhanced Zero-Shot combining Mistral and OpenChat models based on their performance on the OncoDoc2 questions

PETs	Precision (%)	Recall (%)	F1 score (%)	Accuracy (%)
**Enhanced Zero-Shot**	60.16	54.18	56.59	75.57

Abbreviation: PETs, prompt engineering techniques.

**Table 6:** Percentage of compliant recommendations produced by LLM navigations (identical, comparable, different) compared with those issued by MTB clinician navigations

PETs	Model	Identical (%)	Comparable (%)	Different (%)
**Enhanced Zero-Shot**	*Mistral & OpenChat*	3.34	13.33	83.33

Abbreviations: LLM, large language model; MTB, multidisciplinary tumor board; PETs, prompt engineering techniques.

## Discussion

Many studies have evaluated the use of LLMs as CDSSs. A scoping review of 21 studies was recently conducted 18 focusing on studies that used LLMs as CDSSs, and found that the majority of studies (12/21) use LLMs to address clinical cases. The review showed that performance could vary depending on the wording of the questions and the source of the clinical cases. Studies using real patients (5/12) showed lower results (16 to 83%) compared with fictitious patients (58 to 98%). The use of fictitious data was mainly due to confidentiality concerns. Open-source models like Llama, 9 which are recommended in the medical field for data security, were used by only three studies. 19 20 21 Users were *favorable* to using ChatGPT as a CDSS in seven studies, *moderately favorable* in six studies, *neutral* in four studies and *non-favorable* in four others. Despite varying performance, perceptions were generally positive, even for studies with average or low performance. 18

In this work, we wanted to validate the capability of open LLMs to augment an existing CDSS. We started the work with an evaluation to select the best-performing models. The results of evaluating five LLMs on 20 BCPSs (S ^Selection^ ) showed that Mistral and OpenChat stood out based on their performance and low ecological impact compared with the others. OpenChat demonstrated slightly higher accuracy with a range of 69.90 to 70.30% over the two evaluations conducted, compared with 63.50 to 63.90% for Mistral. In terms of carbon impact, OpenChat also showed lower results (0.36 gCO2eq to 0.39 gCO2eq) compared with Mistral (0.48 gCO2eq to 0.54 gCO2eq). Although these criteria guided our selection, it is crucial to critically consider the trade-off between ecological efficiency and clinical accuracy. Our choice reflects a balance between these factors and aligns with the study by Rillig et al, 22 which underscores the importance of energy efficiency to mitigate the ecological impact of LLMs.

Prompt engineering improved LLM's performance, with the enhanced Zero-Shot outperforming both simple Zero-Shot and Zero-Shot CoT PETs. The success of enhanced Zero-Shot in disambiguating questions and guiding binary answers (e.g., “Yes, the patient has breast tumor cancer” and “No, the patient does not have breast tumor cancer”) provided valuable insight into how contextual clarity can improve model responses. Applying the Zero-Shot CoT technique structured models' reasoning process more coherently for some complex questions. However, the decrease in performance with Zero-Shot CoT raised some concerns about potential hallucinations in PET strongly using reasoning. Our hypothesis is that there are many simple questions to which LLMs can easily respond (if the answer is in the context provided), and on adding step-by-step reasoning with Zero-Shot CoT, LLMs may start to hallucinate. For example, for the question on estrogen receptors (OR), the percentage of correct answers when switching from enhanced Zero-Shot to Zero-Shot CoT, dropped from 73.70 to 21.10% for Mistral, and from 60 to 26.30% for OpenChat, suggesting that the added reasoning steps may introduce errors whereas the question is relatively simple. Although results with enhanced Zero-Shot are promising, they reflect specific conditions (when customizing the Zero-Shot prompts) tied to OncoDoc2 decision tree and BCPS dataset. Generalizability to other CDSSs or clinical contexts remains uncertain.

Despite OpenChat showing higher overall accuracy than Mistral (72.47% versus 66.10%), Mistral outperformed OpenChat in specific questions, achieving a higher proportion of results with over 80% accuracy. This suggests that OpenChat is more consistent across different questions, while Mistral excels at handling specific cases. These results can be attributed to OpenChat being pre-trained with varied data and primarily using a Zero-Shot PET, allowing it to respond effectively even with unstructured information, such as the one retrieved in BCPSs. 11 Combining the two models allowed to leverage their respective performances. A similar approach was described by Yu et al, 19 where the authors compared treatment options proposed by four different models and retained the options chosen by at least two LLMs. They found that combining the treatment options of LLMs produced better results than each model individually. In our context, avoiding excessive resource consumption by combining the models based on their performance also showed promising results suggesting that intelligent model-switching is more efficient.

When evaluating LLMs as CDSSs, the combination of Mistral and OpenChat models in enhanced Zero-Shot showed poor results, with 62.68% of the recommendations being different from GS on the S ^Evaluation^ and 83.33% of the recommendations being different from GS on S ^Validation^ . Indeed, when navigating OncoDoc2 decision tree, if the LLM answers one question wrong, this could change the path of the navigation, resulting in the description of a different patient profile leading to different recommendations. Moreover, during manual verification of the results, we found that there were some BCPSs for which the LLM did not provide the correct answer because the elements of response were not present in BCPSs. Therefore, it is important to differentiate errors due to a lack of context and those caused by a poor LLM understanding where the context was correctly given, to better identify and correct the issues.

Another specific problem may have negatively affected the performance of the model combination. Mistral may not respond if the question is not clearly contextualized, which is a problem since a lack of response leads to stopping the navigation and, consequently, to the absence of recommendations.

The outcome of this study reinforces the conclusion that, even with improved instructions, current LLMs are not yet sufficiently reliable to be used as standalone CDSSs. These results are consistent with the conclusions obtained by the review on the use of LLMs as CDSSs. 18 Evaluations on real cases showed poor performance with ChatGPT, a much larger model than those used in this study, in the same breast cancer context. 23 24 25 The three studies presented lower results (respectively 58.8, 70, and 16.05%) than those obtained here, which can be explained by the use of OncoDoc2, whereas the other studies examined LLM's recommendations compared with those of experts.

For future work, exploring specialized models such as CancerLLM 26 or larger LLMs like Mixtral 8 × 7B could improve performance. However, using larger models comes at the cost of environmental impact, as they require more resources. Moreover, simply increasing model size is unlikely to resolve the challenges related to contextual understanding. A more promising direction lies in developing hybrid systems that leverage the complementary strengths of different models. For instance, OpenChat's consistency in general question-answering could be combined with Mistral's ability to handle nuanced cases through a model-switching framework. In addition to hybridization, integrating structured data from electronic health records (EHRs) with LLMs' natural language capabilities could be beneficial and help bridge information gaps. Similarly, incorporating Retrieval-Augmented Generation (RAG) techniques could dynamically enhance LLM's outputs by linking them to external knowledge bases, enabling more contextually accurate and reliable recommendations. The findings of Sahoo et al, 27 which emphasize the divergent behavior of different LLMs to the same prompt, further support the need for model-specific prompt engineering. Optimizing Mistral's prompts to prevent navigation failure in complex decision trees could lead to substantial gains in performance and usability.

## Conclusion

Although this study demonstrates the potential of LLMs to augment CDSSs like OncoDoc2, their current performance remains insufficient for routine clinical use as CDSSs. The majority of the recommendations provided by the models diverge significantly from the gold standard. However, continued research into hybrid models, improved PETs, and the integration of structured data offers promising pathways to enhance LLMs' performance, paving the way for their reliable application in clinical decision support.
