# Supplementary material for: Leveraging Guideline-Based Clinical Decision Support Systems with Large Language Models: A Case Study with Breast Cancer
Source: Methods Inf Med. 2025 Apr 16;63(03-04):85–96. doi: 10.1055/a-2528-4299 (PMC12133322; doi:10.1055/a-2528-4299)
Supplement: Supplementary file 1 — Supplementary Material [file 10-1055-a-2528-4299-s24020011.pdf]

RANG	ID	NIP	NOM	PRENOM	DDN	DATE_RCP	COORD	RESP	COTE
1	736-D	736	A	S	nan	nan	nan	nan	D

DECISION	DECISION_1	DECISION_2	DECISION_3	DECISION_4	DEC_CHIR_IMM	DEC_CHIR	DEC_CHIMIO	DEC_RADIO	DEC_HO
HO neo-adj	HNA	HNA	HO	HO	0	0	0	0	1

CONFORMITE	CAS_PART	PREF_PAT	PREF_RCP	EVOL_PRAT	AUTRE_RAISON	RCP_CHOIX	PROFIL	GROUPE	age-calcule
no	nan	True	nan	nan	nan	False	node70644	PRECHIR	85

TUM_PRESENTE	BILAN_COMPLET	CARCINOME_INVASIF	MICRO_INV	TYPE_LESION_MAM	LESION_MULTIFOCALE_INSITU	LESION_MULTIFOCALE_MICRO_[illegible]	TYPE_IN_SITU	ATCD_CHIR	TYPE_CHIR_MAM
1.0	nan	1.0	0.0	3.0	nan	nan	nan	0.0	nan

CI_TUM	REPRISE-MAST	ATCD_EXP_AX	TYPE_EXP_AX	GS_NEG	CI_GS	REPRISE_CA	CI_CA	CHIR_MAM_IN_SANO	BERGES_CONTACT
0.0	nan	nan	nan	nan	nan	nan	nan	nan	nan

MARGES_INV_NON_ENV	IND_REPR_CHIR_IMM	FAIBLE_VOL_MAM	SUSP_INV	PLEOMORPHE	RES_GS	GANGLIONS	ENV_GG	RH_EVAL	SBR_GRAD
nan	1.0	2.0	nan	nan	nan	nan	nan	nan	nan

RO	RP	MOINS_35	ATCD_CHIMIO_ADJ	CI_TAM	HER2	CHIMIO_ADJ_POSS	CI_ANTHRA	SBR	INDEX_MITO
nan	nan	nan	nan	nan	nan	nan	nan	nan	nan

MENOP_CALC	DECISION_HO	PAT_AGEE	ADENOPATHIE_CLIN	TT_NEOADJ	TYPE_TT_NEOADJ	CNA_COMPLETE	INV_UNIQUE	INV_IN_SITU	TYPE_CHIMIO_NEOADJ
1.0	nan	nan	nan	0.0	nan	nan	1.0	0.0	nan

SG_INFLAM	PAT_OPERABLE	T4_INIT	PROG	CHIMIO_NEOADJ_POSS	TUM_OPERABLE	PLUS_2_N	RECID_LOC	ATCD_MAST	ATCD_CA
nan	1.0	nan	nan	0.0	1.0	0.0	0.0	nan	nan

ATCD_RADIO	CANCER_INFLAM	RADIO_PAROI	F_MAUVAIS_PG	TAILLE_INV	TAILLE_LESION_IN_SITU_PRE_CHI	TAILLE_LESION_IN_SITU_PRE_[illegible]	TAILLE_LESION_IN_SITU_POST_[illegible]	TAILLE_GLOBALE_PRE_CHIR	TAILLE_GLOBALE_PRE_CHIR_3[illegible]
nan	nan	nan	nan	2.0	nan	nan	nan	nan	nan

[illegible]AILLE_GLOBALE_POST_CHIR_	TAILLE_INV_PRE_CHIR	TAILLE_INV_PRE_CHIR_20	TAILLE_CUM_POST_CHIR_40	TAILLE_INV_INIT	TAILLE_INV_ACTUELLE	TAILLE_INV_POST_CHIR_[illegible]	TAILLE_PLUS_GROSSE_INV_POST_CH[illegible]	TAILLE_INV_POST_CHIR_REPRI	Profil_frequent
nan	2.0	nan	nan	nan	nan	nan	nan	nan	1.0

RND	Semaine	Semestre	Ete	Vacances	DEC	RECO	berges-env-in-situ	berges-env-invasif	ind-mast
0.8165162	1	1	0	1	HO	TUM+CA	nan	nan	nan

multif	T-inv-sup-2	GS-meta-ou-cellules	Taille_inv	Taille_globale	Inv_Unique_avec_micro	Pres_micro	Taille_anapath	Score_triple	col_2
nan	nan	nan	nan	nan	nan	nan	nan	nan	nan

Appendix 1 An example of decision representation in OncoDoc's Excel file.

You are a French question-answering system use the context provided to answer the question using only the proposed responses.

CONTEXT
Here is the patient's medical history :
---- CONTEXTE ----

Here is the patient's pathology report (if available):
---- ANAPATH ----

QUESTION
Answer only with the proposed options.
Based on her history, is the N class of the patient's tumor greater than or equal to 2?

OPTIONS
ANSWER 1: Yes.
ANSWER 2: No.

Write only the number of the correct option ('ANSWER 1' or 'ANSWER 2') and DO NOT ADD TEXT

Appendix 2 An example of Zero-Shot prompt, initially in French.

 © 2025. The Author(s).

```
You are a French question-answering system use the context provided to answer the question using only the
proposed responses.

### CONTEXT
Here is the patient's medical history :
---- CONTEXTE ----

Here is the patient's pathology report (if available):
---- ANAPATH ----

#### QUESTION
Answer only with the proposed options.
Based on her history, is the N class of the patient's tumor greater than or equal to 2? This refers to the N in the
TNM classification. N is considered greater than or equal to 2 in cases of fixed ipsilateral lymphadenopathy (N2)
or in the case of ipsilateral mammary involvement (N3).

#### OPTIONS
ANSWER 1: Yes. The patient's tumor class is greater than or equal to 2.
ANSWER 2: No. The patient's tumor class is strictly less than 2.

Write only the number of the correct option ('ANSWER 1' or 'ANSWER 2') and DO NOT ADD TEXT
```

Appendix 3 An example of enhanced Zero-Shot prompt, initially in French.

```
You are a French question-answering system use the context provided to answer the question using only the
proposed responses.

### CONTEXT
Here is the patient's medical history :
---- CONTEXTE ----

Here is the patient's pathology report (if available):
---- ANAPATH ----

#### QUESTION
Answer only with the proposed options.
Based on her history, is the N class of the patient's tumor greater than or equal to 2? This refers to the N in the
TNM classification. N is considered greater than or equal to 2 in cases of fixed ipsilateral lymphadenopathy (N2)
or in the case of ipsilateral mammary involvement (N3).

It can be found in the form TxNy or simply Ny.

#### OPTIONS
ANSWER 1: Yes. The patient's tumor class is greater than or equal to 2.
ANSWER 2: No. The patient's tumor class is strictly less than 2.

Write only the number of the correct option ('ANSWER 1' or 'ANSWER 2') and provide the extract from the
patient's history that supports your choice."
```

Appendix 4 An example of Chain-of-Thoughts prompt, initially in French.

 © 2025. The Author(s).

GS – LLM	TUM	TUM+GS	TUM+CA	MAST	MAST+GS	MAST+CA	GS	CA
TUM	Identical	Comparable+	Comparable-	Comparable-	Comparable-	Different	Different	Different
TUM+GS	Comparable+	Identical	Comparable-	Comparable-	Comparable-	Different	Different	Different
TUM+CA	Different	Comparable-	Identical	Different	Comparable-	Comparable+	Different	Different
MAST	Comparable-	Different	Comparable-	Identical	Comparable+	Comparable-	Different	Different
MAST+GS	Different	Comparable-	Different	Comparable+	Identical	Comparable+	Different	Different
MAST+CA	Different	Different	Comparable-	Different	Comparable-	Identical	Different	Different
GS	Different	Different	Different	Different	Different	Different	Identical	Comparable-
CA	Different	Different	Different	Different	Different	Different	Comparable-	Identical

Legend: TUM = lumpectomy, MAST = mastectomy, CA = axillary clearance, GS = sentinel node

Appendix 5 Comparison of recommendations between large language models (LLMs) and gold standard (GS).

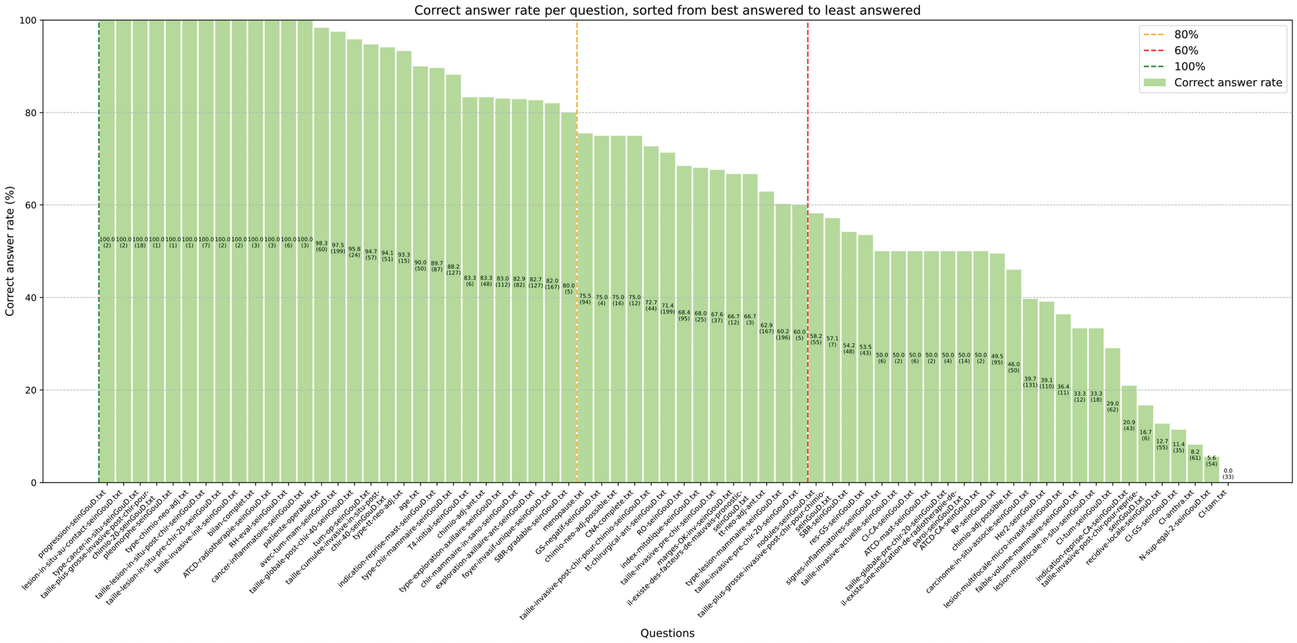

Appendix 6 Distribution of questions sorted by the accuracy of Mistral in enhanced Zero-Shot.

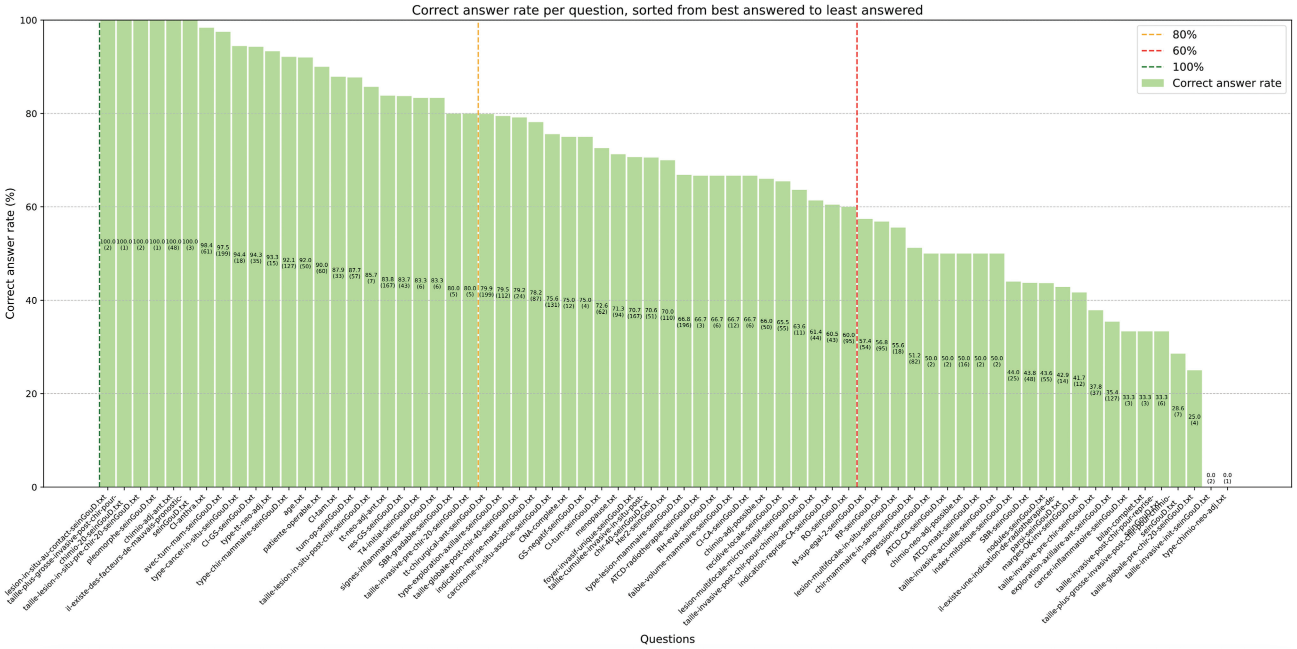

Appendix 7 Distribution of questions sorted by the accuracy of OpenChat in enhanced Zero-Shot.

 © 2025. The Author(s).

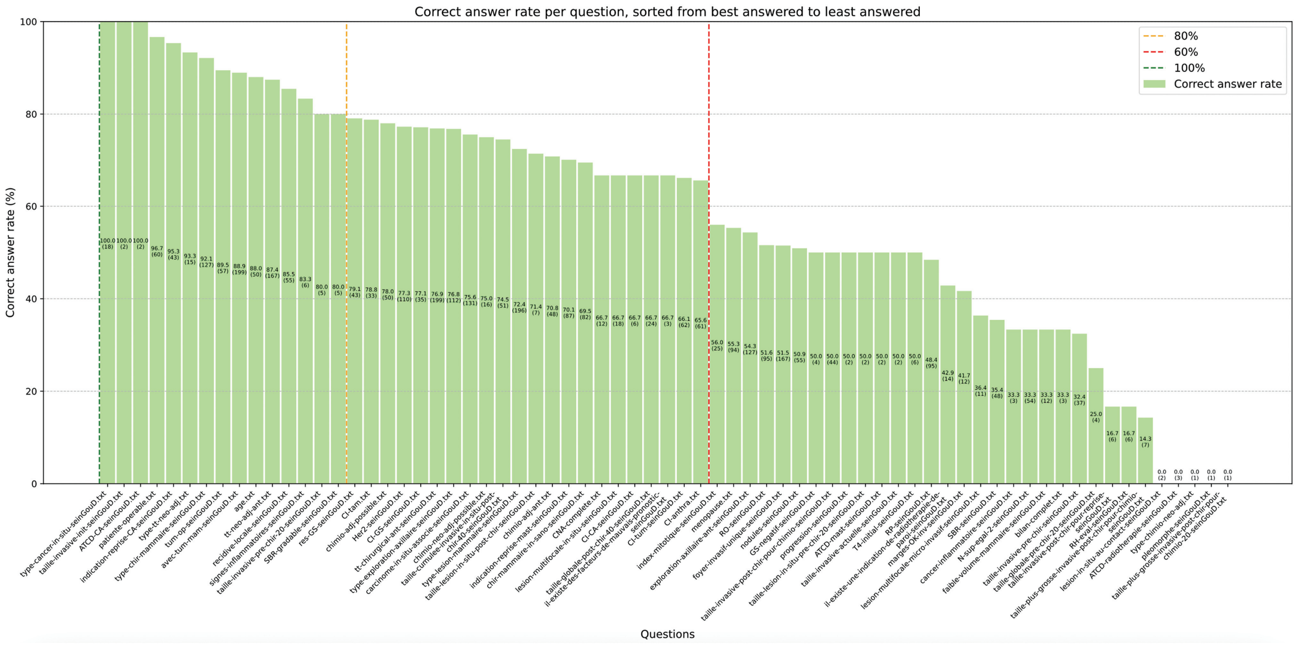

Appendix 8 Distribution of questions sorted by the accuracy of OpenChat model in Zero-Shot CoT.

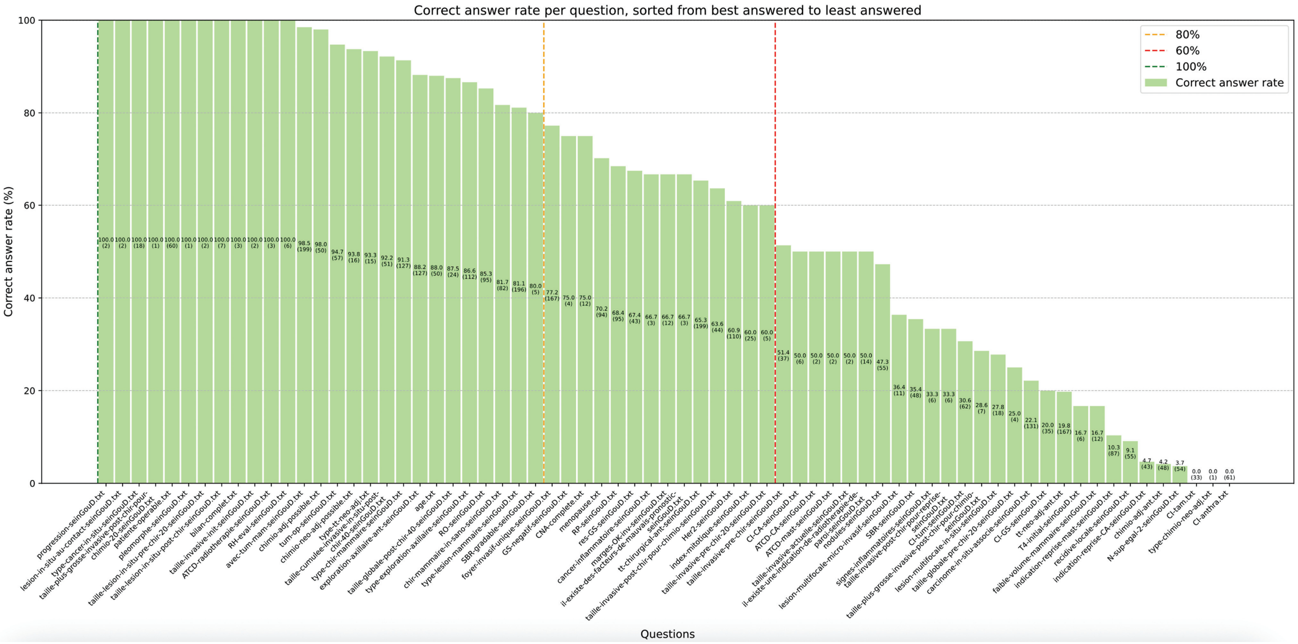

Appendix 9 Distribution of questions sorted by the accuracy of Mistral in Zero-Shot CoT.

 © 2025. The Author(s).
